# The Comparison of Fixed and Flexible Progestin Primed Ovarian Stimulation on Mature Oocyte Yield in Women at Risk of Premature Ovarian Insufficiency

**DOI:** 10.3389/fendo.2021.797227

**Published:** 2022-02-03

**Authors:** Erkan Kalafat, Merve Dizdar, Engin Turkgeldi, Sule Yildiz, Ipek Keles, Baris Ata

**Affiliations:** ^1^ Division of Reproductive Endocrinology and Infertility, Department of Obstetrics and Gynecology, Koc University School of Medicine, Istanbul, Turkey; ^2^ Middle East Technical University, Faculty of Arts and Sciences, Department of Statistics, Ankara, Turkey; ^3^ Department of Obstetrics and Gynecology, Umraniye Teaching and Research Hospital, Istanbul, Turkey; ^4^ ART Fertility Clinics, Dubai, United Arab Emirates

**Keywords:** PPOS, anti-Mullerian hormone, cryopreservation, elective, mature oocyte, yield

## Abstract

While gonadotrophin releasing hormone (GnRH) antagonists have been the standard of pituitary suppression during ovarian stimulation for ART, progestin primed ovarian stimulation (PPOS) has emerged as an alternative. Progestins can be started simultaneously with gonadotrophins (fixed PPOS) or later in the cycle depending on follicle growth (flexible PPOS). However, the flexible and fixed PPOS regimens have not been directly compared as of yet. This was a retrospective cohort study including women with diminished ovarian reserve who underwent oocyte cryopreservation. All women underwent ovarian stimulation with a fixed 300 IU daily dose of FSH. The primary outcome was the number of MII oocyte retrieved per cycle. Secondary outcome measures included the incidence of premature LH surge (>10ng/mL) and number of follicles larger than 14mm on the day of maturation trigger. During the screening period 2 out of 97 cycles were cancelled before oocyte retrieval, one in each group yielding an overall cancelation rate of 2%. Among women who had oocyte retrieval, 65 underwent flexible and 30 fixed PPOS. At baseline women on fixed and flexible PPOS had similar age (mean difference: -2.17 years, 95*% CI:* -4.46 to 0.11) and serum AMH levels (mean difference: 0.10 ng/mL, 95% CI: -0.24 to 0.47). Slight imbalances between the groups were rectified with propensity score matching using age and AMH levels. The incidence of premature LH surge (RR: 1.47, 95% CI: 0.51 – 5.27, p = 0.50), follicle count larger than 14mm on hCG day (RR: 1.14, 95% CI: 0.93 – 1.42, p = 0.22), number of MII oocytes retrieved (RR: 0.95, 95% CI: 0.79 – 1.15, p = 0.61) were similar between flexible and fixed PPOS. The rate of no oocyte retrieval was same between the groups (0.0% both) but no formal estimation was possible. Flexible and fixed PPOS regimens had no appreciable differences regarding MII oocyte yield and the incidence of premature LH surges. Cycles without oocyte retrieval were rare in both groups and ultrasonographic parameters of gonadotropin response were similar. Our study suggests the performances of either progestin regimen are comparable in this group of women.

## Introduction

Stimulation of multifollicular growth with gonadotropins and pituitary suppression to prevent premature luteinizing hormone (LH) surge and ovulation are employed to increase the effectiveness of each egg freezing cycle. While gonadotrophin releasing hormone (GnRH) antagonists have become the standard of pituitary suppression, progestin primed ovarian stimulation (PPOS) has emerged as an alternative (1). The lower cost of progestin combined with ease of use (oral application vs. injections) make it an appealing option for ovarian stimulation cycles when a fresh embryo transfer is not intended (e.g., oocyte freezing,oocyte donors, hyper-responders, preimplantation genetic testing plans etc.). Several studies have shown that PPOS yields a similar number of metaphase-II (MII) oocytes compared to GnRH antagonist cycles when progestin is started simultaneously with gonadotrophins and continued until the day of ovulation trigger (2, 3). An alternative approach dubbed as the flexible PPOS, akin to flexible GnRH antagonist protocols, where progestin is started later in the cycle, based on leading follicle size or serum estradiol and/or LH levels, has also been used for pituitary suppression. Observational studies have shown flexible PPOS yields similar MII oocytes with GnRH antagonists in high- or low- responders alike (4, 5). However, whether outcomes of the flexible and fixed PPOS regimens are different is unknown. In this observational study, we compared flexible and fixed PPOS regimens in women with diminished ovarian reserve who were undergoing elective oocyte cryopreservation.

## Methods

This was a retrospective cohort study using anonymized patient data. All women were treated at the Assisted Reproduction Unit of the Koc University Hospital between 07/2018 and 08/2021. Women undergoing ovarian stimulation for oocyte cryopreservation were screened for eligibility. Local regulations only allow nulliparous women with diminished ovarian reserve [as determined by low AMH levels, diminished antral follicle count (AFC) or advanced female age] or at high-risk of premature ovarian insufficiency (family history of early menopause) to freeze their oocytes in the absence of a medical indication (i.e., pending gonadotoxic chemotherapy or radiotherapy). All eligible women either had diminished ovarian reserve, defined as a serum AMH level of <1.5 ng/ml or AFC< 10, or had family history of premature ovarian insufficiency (5). Inclusion criteria were regular menstrual cycles between 21 – 35 days, undergoing PPOS with either a flexible or fixed progestin regimen. Women undergoing GnRH antagonist cycles, with missing AMH values or with a medical indication for fertility preservation were excluded.

Ovarian pathology was excluded with a baseline ultrasound scan on the 2^nd^ or 3^rd^ day of menstruation. All women underwent ovarian stimulation with a fixed 300 IU daily dose of recombinant follicle stimulating hormone (rFSH) (Gonal-f, Merck, Switzerland). Fixed progestin regimen group were started on 10 mg/daily medroxyprogesterone acetate (MPA) p.o. simultaneously with rFSH. In the flexible PPOS, MPA 10 mg/day p.o. was started when the leading follicle **≥** 14 mm or serum estradiol (E_2_) >200 ng/mL, whichever came first. In both protocols MPA was continued until (including) the day of ovulation trigger. Oocyte maturation was triggered with 250 mcg of recombinant human chorionic gonadotropin injection (hCG, Ovitrelle, Merck Serono, Switzerland), when the leading follicle size was greater than 17 mm, followed by oocyte retrieval 36 hours later. Cumulus corona complexes were denuded 2 – 4 hours later and metaphase-II oocytes were vitrified.

The primary outcome measure was the number of MII oocyte retrieved per cycle. Secondary outcome measures included premature LH surge (>10 ng/mL) and number of follicles larger than 14 mm on the day of maturation trigger.

### Statistical Analysis

Continuous variables with a normal distribution were defined with mean and standard deviation, while skewed distributions were defined with median and 25^th^ – 75^th^ percentiles. Age and AMH levels were identified as potential confounders and groups were checked for imbalances regarding these variables with propensity scores. Then groups were matched on age and AMH levels with exact matching on AMH levels, which were rounded to the nearest one decimal place to help finding matches. Replacement was allowed if no unique alternative could be found for an individual. After balancing the propensity score of both groups, the outcomes were compared between flexible and fixed PPOS groups with mixed effect generalized models using match and patient identifier (repeat cycles) as random effects. Log-binomial link function was used for binary outcomes (premature LH surge) and log-Poisson link function was used for count data (MII oocyte count, follicle count). Outcomes were reported as risk ratios with 95% confidence intervals. A sample size of ~90 women with 1:2 allocation was planned as it allowed for the estimation of 25% change ( ± 1 oocyte) in MII oocyte count or more with adequate power (alfa: 0.05; beta:0.80) and with a median MII oocyte yield of 4. Violin plots were used for graphical representation of the results. All analyses were conducted with R for Statistical Computing Software (v.4.0.4).

## Results

**Figure 1:**
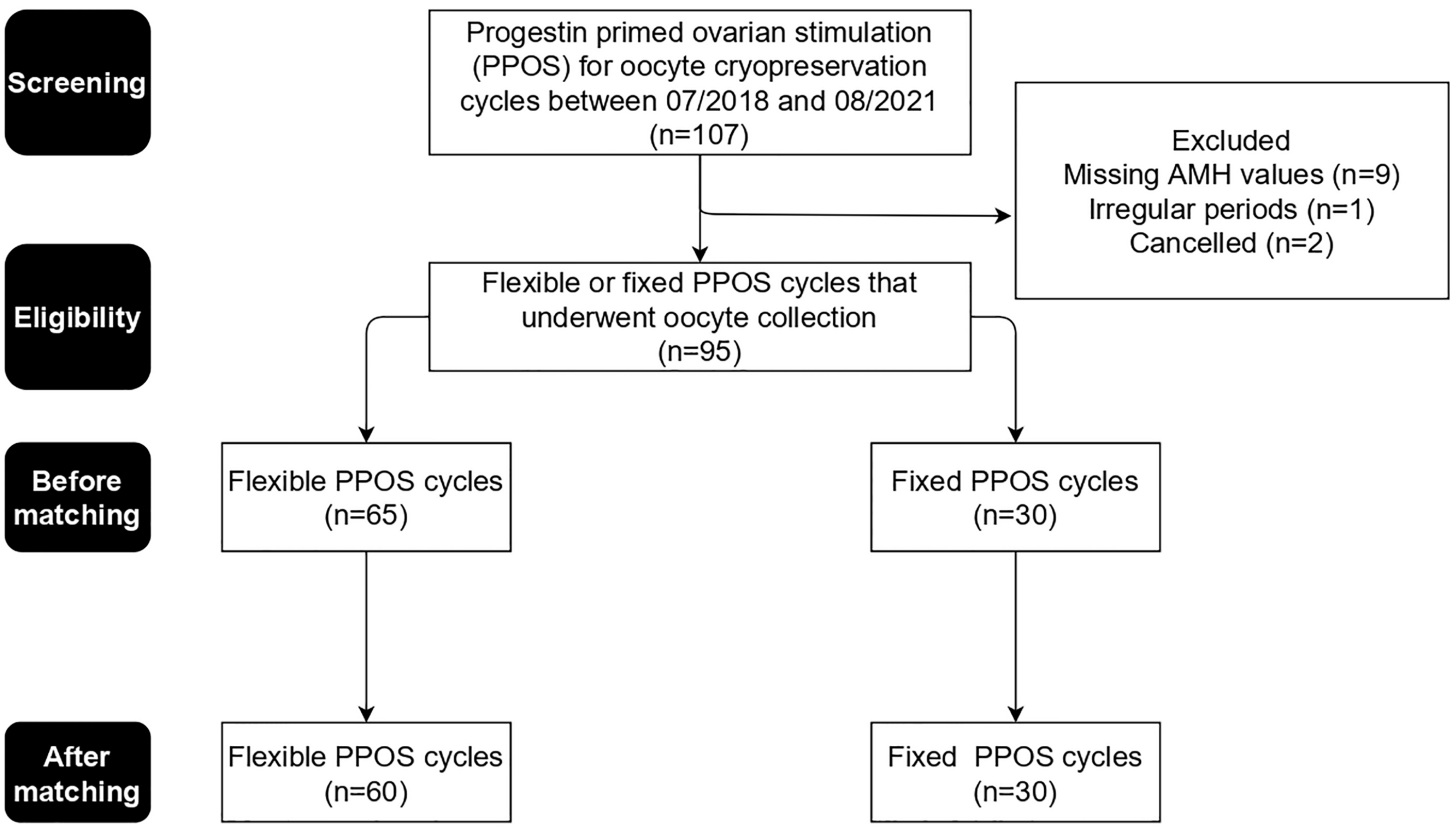
Study enrolment diagram.

During the screening period, there were 107 PPOS cycles of 82 women for oocyte cryopreservation. All women were nulligravid. Nine cycles were excluded due to missing AMH values, one was excluded due to irregular periods (**Figure 1**). Two of the remaining 97 PPOS cycles were cancelled before oocyte retrieval, one in each group, yielding an overall cancelation rate of 2%.

Among women who had oocyte retrieval, 65 with flexible and 30 with fixed PPOS. At baseline women on fixed and flexible PPOS had similar age (mean difference: -2.17 years, 95*% CI:* -4.46 – 0.11, P = 0.062), BMI (mean difference: 0.90 kg/m^2^, 95% CI: -0.55 – 2.37, P = 0.217) and serum AMH levels (mean difference: 0.10 ng/mL, 95% CI: -0.24 – 0.47, P = 0.524). Slight imbalances between the groups were rectified with propensity score matching using age and AMH levels (**Figure S1**). After matching the groups were better balanced regarding age and baseline AMH levels with mean difference effect size less than 0.05 (Cohen’s D) (**Table 1**).

**Table 1:** Baseline characteristics of oocyte freezing cycles before and after propensity score matching.

Before matching	Fixed PPOS Cycles (n = 30)	Flexible PPOS Cycles (n = 65)	MD (95% CI)	P
Age in years	35.9 ± 4.90	38.1 ± 5.77	-2.17 (-4.46 – 0.11)	.06
Body-mass index in kg/m^2^	22.2 ± 3.39	21.3 ± 3.07	0.90 (-0.55 – 2.37)	.22
AMH levels in ng/mL	1.14 ± 0.86	1.03 ± 0.64	0.10 (-0.24 – 0.47)	.52
***After propensity score matching***	**Fixed PPOS Cycles (n = 30)**	**Flexible PPOS Cycles (n = 60)**	**MD (95% CI)**	***P***
Age in years	35.9 ± 4.90	36.0 ± 5.09	-0.10 (-2.32 – 2.12)	.93
Body-mass index in kg/m^2^	22.3 ± 3.39	21.0 ± 3.54	1.28 (-0.26 – 2.82)	.10
AMH levels in ng/mL	1.15 ± 0.86	1.13 ± 0.62	0.02 (-0.33 – 0.38)	.90

PPOS, progestin primed ovarian stimulation; MD, mean difference; AMH, anti-Mullerian hormone; CI, confidence interval.

**Figure 2:**
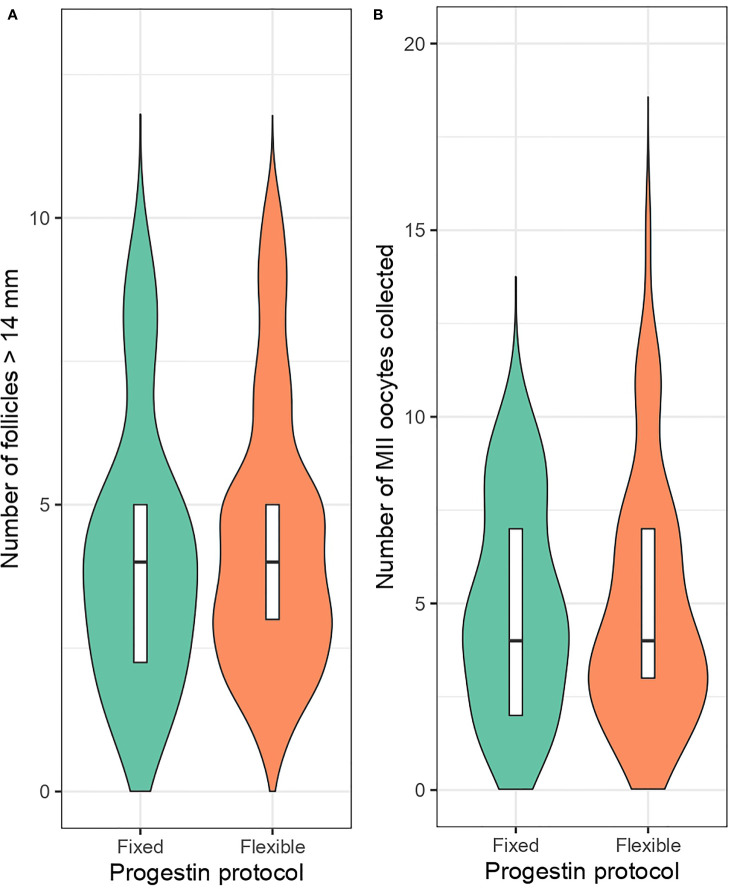
Number of follicles larger than 14mm on maturation trigger day **(A)** and retrieved metaphase-II oocyte count **(B)** in fixed and flexible progestin regimen groups.

Analysis of the matched cohort with mixed-effects regression models showed no significant differences between flexible and fixed progestin protocols regarding the incidence of premature LH surge (RR: 1.47, 95% CI: 0.51 – 5.27, p = 0.50), follicle count larger than 14mm on hCG day (RR: 1.17, 95% CI: 0.95 – 1.45, p = 0.14), number of MII oocytes retrieved (RR: 0.94, 95% CI: 0.78 – 1.14, p = 0.56) (**Table 2**). The rate of no oocyte retrieval was same between the groups (0.0 vs. 0.0%, fixed vs. flexible) but no formal estimation was possible. The median counts of follicles larger than 14mm on hCG day (median: 4.0 vs. 4.0, fixed vs. flexible PPOS, respectively) and number of collected MII oocytes (median: 4.5 vs. 4.0, fixed vs. flexible PPOS, respectively) along with distribution characteristics of each variable are presented as violin plots in **Figure 2**.

**Table 2:** Outcome of fixed and flexible progestin regimens on ovulation induction outcomes.

Variables	Fixed PPOS Cycles (n = 30)	Flexible PPOS Cycles (n = 60)	RR (95% CI)	P
Duration of stimulation in days	8 (7 – 9)	8 (7 – 10)	0.97 (0.89 – 1.05)	.48
Total gonadotropin consumption in IU	2400 (2100 – 2700)	2400 (2100 – 3000)	0.97 (0.89 – 1.05)	.48
LH surge (>10) prior to hCG	4 (13.3%)	12 (20.0%)	1.47 (0.51 – 5.27)	.50
Follicle count >14mm on hCG day	4.0 (2.25 – 5.0)	4.0 (2.0 – 7.0)	1.17 (0.95 – 1.45)	.14
Number of MII oocytes	4.5 (2.25 – 7.75)	4.0 (2.0 – 7.0)	0.94 (0.78 – 1.14)	.56
No oocyte retrieved	0 (0.0)	0 (0.0)	NE	–

LH, luteinizing hormone; PPOS, progestin primed ovarian stimulation; RR, risk ratio; NE, not estimable; MII, metaphase-II; hCG, human chorionic gonadotrophin.

## Discussion

In the present sample flexible and fixed PPOS had no appreciable differences regarding MII oocyte yield and the incidence of premature LH surges. Cycles without oocyte retrieval were rare in both groups and ultrasonographic parameters of response to stimulation were similar.

Inclusion of all cases available within a specified time frame to limit selection bias, matching for important confounders by propensity score, using a fixed dose of the same gonadotrophin for all patients, and all treatment decisions being taken by a single physician (BA) are the strengths of the study. The outcomes, (e.g., metaphase two oocytes, etc.) were not subject to measurement bias depending on exposure (fixed vs. flexible PPOS). All these points put our estimates at low to moderate risk of bias according to observational study bias assessment tool, ROBINS-I (6). While the sample size can be regarded as a limitation of our study, it is powered for the estimation of a ≥25% change, which corresponds to the smallest possible change in the unit of analysis (i.e., one MII oocyte with a median oocyte yield of four). The literature is limited on this topic and our contribution could be combined into pooled estimates of systematic reviews in the future. Failure to report pregnancy or live birth rates is a limitation, yet currently available data does not suggest that oocyte competency to reach live birth is affected by pituitary suppression protocols (1). In women with poor ovarian reserve, live birth rate per stimulation cycle is usually low and an unattainably high number of participants are required to demonstrate any small differences that can be brought about by stimulation protocols as statistically significant. Thus, many studies involving women with poor ovarian reserve, rely on oocyte yield as a measurable intermediate outcome to direct clinical practice. Yet, the observations from the present study are only generalizable to women with decreased ovarian reserve, who may have different follicular growth dynamics than women with normal or high ovarian reserve, e.g., more inclined to premature ovulation (4).

A 24-year-old girl who had a unilateral endometrioma of 10 cm and a serum AMH level of 0.35 ng/ml had a cycle cancellation after 18 days of stimulation with fixed PPOS when there were no follicles >16 mm and her serum estradiol level stagnated around 180 pg/ml. Her serum FSH levels ranged between 38 and 77 IU/L during stimulation. It seems unlikely that pituitary suppression protocol prevented ovarian response. The woman with premature ovulation in the flexible PPOS protocol was 35 years old, had a serum AMH level of 0.47 ng/ml. She was started MPA on the 5^th^ day of the stimulation, when the leading follicle was 14 mm, two days later her LH levels was increased and estradiol was decreased despite growing follicle size, and on the 9^th^ day of stimulation her progesterone level rose to 2.76 ng/ml, estradiol had risen again with declining LH while there were three follicles measuring 18, 17 and 16 mm. It was decided that the ovulation process had started and the cycle was cancelled. Subsequently she underwent four other cycles and also had the ovulation process started before ovulation trigger in the second and third cycles. Her serum estradiol levels dropped, progesteron levels started to rise and LH levels fluctuated before pituitary suppression was started on the 5^th^ day of stimulation when the leading follicle was 15 mm in the second cycle. She was given hCG that evening but egg retrieval was cancelled due to follicle rupture in the morning of scheduled procedure. Her third cycle was a fixed PPOS regimen and her serum estradiol level decreased to 484 pg/ml from 509 pg/ml between the sixth and eight days of stimulation without an increase in LH and progesterone. A fixed PPOS protocol was combined with a flexible GnRH antagonist protocol in her fourth and fifth cycles which yielded 14 metaphase two oocytes in total. Repeated premature ovulations with different pituitary suppression protocols make us think that she had a tendency for premature ovulation.

Only few studies have reported on the results of flexible PPOS so far (4, 5, 7). When compared with the flexible antagonist protocol, the flexible PPOS seemed to be associated with increased oocyte yield in high responders (4). In women with diminished ovarian reserve conflicting results were reported (8). Durdag et al. reported a high-rate of premature ovulation in women with poor ovarian reserve (11.5 vs. 0.0%) with flexible PPOS compared to antagonist cycles. However, the number of collected MII oocytes were similar despite the increased rate of premature ovulation, which corroborates the findings of Turkgeldi et al. (5, 7)

To the best of our knowledge, the present study is the first to compare fixed and flexible PPOS protocols, and results suggest an overall low rate of premature ovulation and similar oocyte yield with both protocols. Indirectly suggesting they can be as effective as the flexible GnRH antagonist protocol in this group of women.

Although we tried to reduce confounding effects by propensity score matching, randomized trials would still be preferable. Ideally, trials in women with different categories of ovarian reserve should be performed and report on measures of oocyte competence, e.g., fertilization, blastulation, pregnancy and live birth rates.

Mature oocyte yield was similar with either flexible or fixed PPOS in women with diminished ovarian reserve. Cycle cancelation and no oocyte retrieval were rare in both groups and there was no increased risk of premature LH surge.

## Data Availability Statement

The datasets presented in this article are not readily available because of institutional data protection regulations. Requests to access the datasets should be directed to Koc University Hospital. Requests to access the datasets should be directed to barisata@ku.edu.tr.

## Ethics Statement

The studies involving human participants were reviewed and approved by Koc University Ethics Review Board. Written informed consent for participation was not required for this study in accordance with the national legislation and the institutional requirements.

## Author Contributions

Conception & Design (BA and EK), Data acquisition (MD and IK), Analysis (EK), Drafting (BA and EK), Critical revision (BA, EK, ET, SY, and IK), Final approval (BA, EK, ET, SY, and IK). All authors contributed to the article and approved the submitted version.

## Conflict of Interest

The authors declare that the research was conducted in the absence of any commercial or financial relationships that could be construed as a potential conflict of interest.

## Publisher’s Note

All claims expressed in this article are solely those of the authors and do not necessarily represent those of their affiliated organizations, or those of the publisher, the editors and the reviewers. Any product that may be evaluated in this article, or claim that may be made by its manufacturer, is not guaranteed or endorsed by the publisher.

## Supplementary Material

Supplementary Figure 1 Propensity score histograms of fixed (treated) and flexible (control) progestin groups before and after the matching.
