# Supplementary figures and images for: The Comparison of Fixed and Flexible Progestin Primed Ovarian Stimulation on Mature Oocyte Yield in Women at Risk of Premature Ovarian Insufficiency
Source: Front Endocrinol (Lausanne). 2022 Feb 3;12:797227. doi: 10.3389/fendo.2021.797227 (PMC8850276; doi:10.3389/fendo.2021.797227)

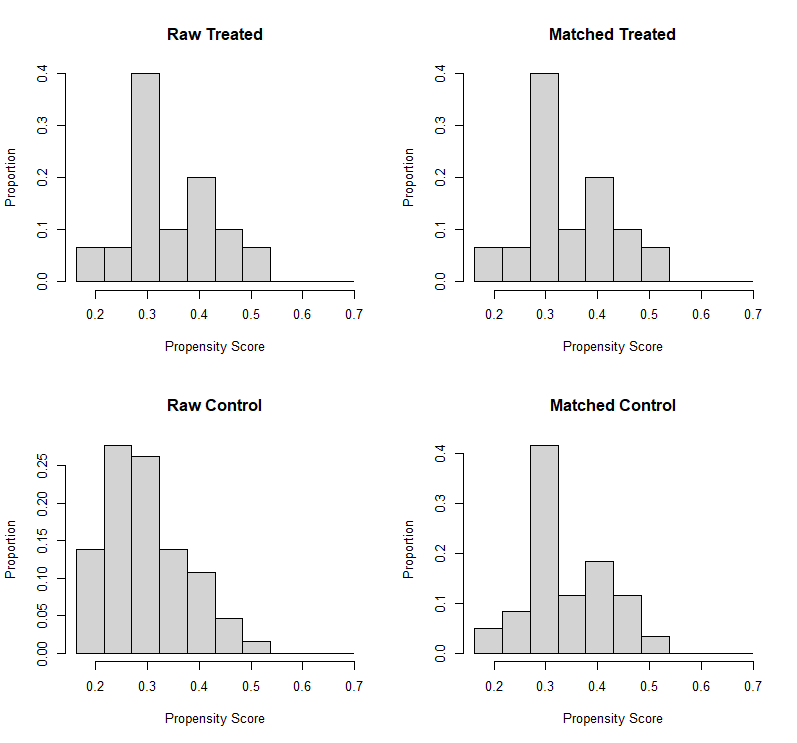

Supplement: Supplementary Figure 1 — Propensity score histograms of fixed (treated) and flexible (control) progestin groups before and after the matching. [file Image_1.tiff]
